# Single-pixel imaging of dynamic objects using multi-frame motion estimation

**DOI:** 10.1038/s41598-021-83810-z

**Published:** 2021-04-08

**Authors:** Sagi Monin, Evgeny Hahamovich, Amir Rosenthal

**Affiliations:** Department of Electrical Engineering, Technion – Israel Institute of Technology, 32000 Haifa, Israel

**Keywords:** Imaging techniques, Optics and photonics, Imaging and sensing

## Abstract

Single-pixel imaging (SPI) enables the visualization of objects with a single detector by using a sequence of spatially modulated illumination patterns. For natural images, the number of illumination patterns may be smaller than the number of pixels when compressed-sensing algorithms are used. Nonetheless, the sequential nature of the SPI measurement requires that the object remains static until the signals from all the required patterns have been collected. In this paper, we present a new approach to SPI that enables imaging scenarios in which the imaged object, or parts thereof, moves within the imaging plane during data acquisition. Our algorithms estimate the motion direction from inter-frame cross-correlations and incorporate it in the reconstruction model. Moreover, when the illumination pattern is cyclic, the motion may be estimated directly from the raw data, further increasing the numerical efficiency of the algorithm. A demonstration of our approach is presented for both numerically simulated and measured data.

## Introduction

Single-pixel imaging (SPI) represent an imaging methodology in which the structure of objects is determined using a single detector and a set of spatially modulated illumination patterns that are sequentially projected onto the object. The signals measured by the detector represent the projections of the image onto the basis span by the illumination patterns. To recover the image from the measured data, a computational technique is used. SPI is an attractive approach in fields where camera technology is limited and it has found numerous applications in the last decade: Terahertz imaging^1^, LIDAR^2^, compressed sensing^3^, single pixel telescope^4^, optoacoustic imaging^5^, multispectral imaging^6^, 3D imaging^7,8^, imaging through scattering medium^9,10^, fluorescence microscopy^11^, multi-photon imaging^12–14^ and image encryption^15,16^.

One of the attractive features of SPI is its compatibility with the theory of compressed sensing (CS), exploiting image sparsity in order to perform the computational reconstruction with fewer measurements than prescribed by the Nyquist criterion^17^. One of the key parameters that determine the performance of CS algorithms is the choice of patterns projected on the object, with various types of patterns proposed, including Fourier basis^18^, wavelet trees^19^, optimized ordering of Hadamard basis^20^, and basis optimized by deep learning^21^.

One of the major limitations of SPI is its low imaging speed, which is a result of its sequential data acquisition and experimental apparatus. Conventionally, digital-mirror devices (DMDs), operating at rates of up to 22000 binary patterns per second, are used to spatially modulate the light. Recently, a method for faster spatial modulation in SPI has been demonstrated, e.g. using arrays of light-emitting diodes^22^. Despite these advancements, SPI remains highly sensitive to motion artifacts, as it requires that the object remains static during the acquisition of a single image. In contrast to camera-based imaging, object motion in SPI leads not only to image smearing, but additionally to reconstruction artifacts and loss of image contrast, which occur even in the presence of relatively small motion of few pixels.

In recent years, several approaches have been developed to improve the compatibility of SPI with imaging dynamic objects. In Refs.^23,24^, adaptive illumination patterns were used to increase the imaging speed only in areas of the image in which motion was detected, allowing for lower imaging rates in the static portions of the image. Adaptive illumination patterns with spatially varying resolution were used to increase the resolution only in areas of the image where motion was detected, allowing for a reduction in the total number of pixels and thus enabling higher imaging rates. The main downside of these approaches is reliance on real-time computation to adapt the DMD projected patterns. In Ref.^25^, motion estimation was performed during the capture of a single frame, where the estimated motion of the object was incorporated into the reconstruction algorithm. While this approach performed well on both simulated and measured data, it suffers from several limitations. First, it assumes a known motion model (e.g. linear translation, rotation) with constant velocity, and cannot infer the model from the data, thus limiting its application in more complex scenarios, e.g. imaging of an accelerating object. Second, to estimate the motion parameters, an iterative optimization algorithm was employed, requiring several reconstructions and motion shifts to sampling patterns to be performed. For even modest size images of 128x128, both procedures are time-consuming. Third, the estimation of the motion parameters was based on assumptions on the image properties, potentially limiting the technique in some image classes.

In this paper, we develop a new approach for SPI in dynamic scenarios in which the image, or parts thereof, moves over a single trajectory during data acquisition. We restrict our analysis to 2D images, assuming that all motion occurs within the imaging plane. In contrast to Ref.^25^, we perform motion estimation between subsequent frames, rather than within the same frame, leading to significant reduction in complexity. While our algorithm may be applied to any type of trajectory, it assumes that within a single frame the motion is approximately linear. Once the motion in the image has been determined, it is incorporated into the reconstruction algorithm, similarly to Ref.^25^. Further improvement in the reconstruction efficiency is achieved by using a cyclic sampling basis, which enables motion estimation in the measurement space and efficient incorporation of the estimated motion into the imaging model. We successfully demonstrate our approach on both simulated and measured data for two types of motion: global motion of the entire image and local motion of only parts of the image.

## Methods

### The mathematical model of SPI

For an image $\mathbf {U}[p,q]$ with *P* rows and *Q* columns, the imaging process of SPI is comprised of two stages. The first stage involves illuminating the object with a set of spatially varying patterns and collecting the remitted light. Mathematically, this procedure may be described by the following matrix equation:

1 $$\begin{aligned} \mathbf {b} = \mathbf {S}\mathbf {u}, \end{aligned}$$

where $\mathbf {u}$ is a row stacked vector with length $N=P \times Q$, formulated as $\mathbf {u}[q + pQ] = \mathbf {U}[p,q]$ containing the intensity values of each image pixel, $\mathbf {b}$ is a row stacked vector of the measurement values with length *M*, and $\mathbf {S}$ is the sampling matrix, where each row represents one of the projection patterns.

The second stage in SPI is the reconstruction of the image. When the matrix $\mathbf {S}$ is square and well-conditioned, the inversion of Eq. (1) may be readily achieved by calculating the inverse of $\mathbf {S}$:

2 $$\begin{aligned} \mathbf {u} = \mathbf {S}^{-1}\mathbf {b}. \end{aligned}$$

For a general matrix $\mathbf {S}$ with $M<N$, i.e. with fewer measurements than the number of pixels, the reconstruction may be formulated as an optimization problem:

3 $$\begin{aligned} \mathbf {u^*} = \text {argmin } ||\mathbf {u}||_2 \text { subject to } \mathbf {b} = \mathbf {S}\mathbf {u}, \end{aligned}$$

where a closed-form solution exists, known as pseudo-inverse: $\mathbf {u^*} = [\mathbf {S}^T \mathbf {S}]^{-1} \mathbf {S}^T b$.

In CS, the image is assumed to have some sparse representation, and a new optimization problem is formulated with regularization, trying to estimate the sparse representation. One of the common cost functions used for regularization is the total-variation (TV) functional, for which the following optimization problem is solved:^26^:

4 $$\begin{aligned} \mathbf {u^*} = \text {argmin } ||\mathbf {u}||_{TV} \text { subject to } ||\mathbf {b} - \mathbf {S}\mathbf {u}||^2 < \sigma , \end{aligned}$$

where TV is described by the derivatives of the image $\mathbf {u_{TV}} = \sum _{i=1}^N (\partial u / \partial x + \partial u/\partial y )$, and $\sigma$ is some bound on the noise. Solving Eq. (4), is a well studied problem with many available solvers, .e.g TVAL3^27^, used in this work.

When the imaged object or scene is not stationary, but rather dynamic, the acquisition model described in Eq. (1) is no longer valid. Instead of a single image $\mathbf {u}$, each pattern illuminates a slightly modified version of the image, denoted by $\mathbf {u}_i$, and the acquisition is described by:

5 $$\begin{aligned} \mathbf {b}[i] = \mathbf {s}_i^T \mathbf {u}_i \text {, for } 1 \le i \le N, \end{aligned}$$

where $\mathbf {s}_i^T$, $\mathbf {u}_i$ and $\mathbf {b}[i]$ are the $i^{th}$ row of the sampling matrix, the $i^{th}$ image and $i^{th}$ measurement respectively. For the forward model described in Eq. (5) with $\mathbf {u}_i \ne \mathbf {u}_j$ for some *i* and *j*, the inversion operation of Eq. (2) is no longer exact, and may lead to significant artifacts. A common method to reconstruct the image is to use CS and solve Eq. (4), where only a small subset of measurements are used. In this scenario we have a trade-off between artefacts caused from using a small set of measurements and artefacts caused by the dynamic nature of the scene.

### Sampling matrix

The sampling matrix $\mathbf {S}$, representing the illumination patterns, plays an important rule in SPI. One common sampling matrix is the Hadamard matrix, a binary matrix with “1” and “-1” as its entries^26^. In this work, we use the S-matrix, a binary matrix with elements of “0” and “1”^28^. S-matrices have a closed form inversion $S^{-1} = \frac{2}{N+1} (2S^T - J)$, where *N* is the order of the matrix, and *J* is matrix of ones. One of the advantages of S-matrix is that under some conditions, it has a cyclic structure, where every row of the matrix is a cyclic shift of the previous row. This cyclic structure enables detecting motion of objects directly in the measurement space. In this work, we use the Twin-Prime algorithm^28^ to construct the S-matrix, where the matrix size is a multiplication of two consecutive prime numbers *P* and *Q*. An example for an S-matrix is given in supplementary Fig. S1.

Since our sampling matrix is cyclic, the sampling procedure may be written as a cyclic convolution between the image and the first row of the sampling matrix^29^:

6 $$\begin{aligned} \mathbf {B} = \tilde{\mathbf {S}} \circledast \mathbf {U}, \end{aligned}$$

where $\tilde{\mathbf {S}}$ is the convolution matrix, $\circledast$ is a cyclic convulsion operator, and $\mathbf {B}$ is a matrix of the sampled measurements of size $P \times Q$, which we term as projection space. The matrix $\mathbf {B}$, can also be formulated by solving Eq. (1) and rearrangement, using $\mathbf {B}[p,q] = \mathbf {b}[q + p*Q]$.

**Figure 1:**
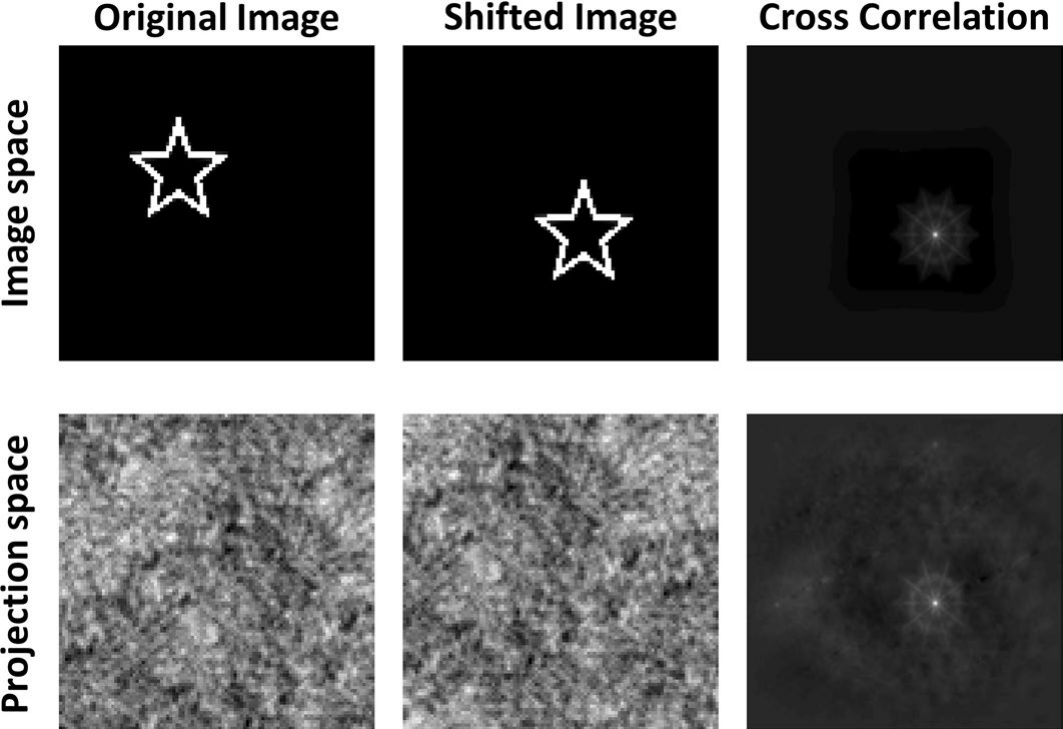
For a cyclic sampling matrix, spatial translation of the image results in the same translation in projection space. The top row shows an image of a star, a shifted version of that image, and the corresponding cross-correlation between the two images. On the bottom row, the first two panels show the corresponding projection images, calculated by applying Eq. 1 on the respective images on the top row. While the spatial translation is difficult to observe visually in the projection images, it is readily detected in the cross correlation between the two images, shown in the third panel in the second row.

Since the projection operation is equivalent to performing cyclic convolution between the image $\mathbf {U}$ and the sampling matrix $\tilde{\mathbf {S}}$ Eq. (6), some of the spatial properties of the image are preserved in projection space $\mathbf {B}$. In particular, since $\tilde{\mathbf {S}}$ is a constant matrix, a circular shift in the image matrix $\mathbf {U}$ leads to a circular shift in the projection-data matrix $\mathbf {B}$. As we show in the Supplementary Information, when edge effects are ignored, the implication of Eq. (6) is that a shift in image space, over each of its two axes, leads to a similar shift in projection space, as illustrated in Fig. 1. Accordingly, motion may be estimated directly from the projection data by comparing the projection images of two subsequent frames.

### SPI with subsequent frames

In this work, we assume that the object is imaged continuously and that *K* sets of $\mathbf {b}$ are measured with the same square matrix $\mathbf {S}$ Eq. (1), leading to a measurement vector, $\mathbf {b}_{\text {tot}}$ with length $K \cdot N$. To detect motion, we compare the reconstructions obtained from subsequent frames. The simplest method to compare between frames, is to reconstruct *K* different frames using Eq. (2), each derived from a different set of full measurements, denoted by $\mathbf {b}_k$. While this approach is ideal in the case of linear motion at a constant velocity, as we show in the Results, when the velocity varies during the acquisition time of a frame, it is beneficial to perform more than *K* reconstructions.

We propose two approaches for increasing the number of reconstructions beyond *K*. In the first approach, instead of performing a single image reconstruction from each full-measurement vector $\mathbf {b}_k$, each vector $\mathbf {b}_k$ is divided into *I* subsets with a length of $O=N/I$, denoted as $\mathbf {b}_{k,i}$. Approximate reconstruction is then performed on each of the *I* subsets using Eq. (3) to reconstruct images $\mathbf {u}_{k,i}$ leading to $I \cdot K$ approximate images. While the *I* partial reconstructions are expected to manifest higher noise levels due to missing data, they experience less motion than the full reconstruction, obtained from *N* samples. In the second approach, we use the repetition of the sampling matrix $\mathbf {S}$. Since the illumination patterns are repeated, each series of *N* consecutive elements in $\mathbf {b}_{\text {tot}}$ can be used to reconstruct a single image via Eq. (2). Hence, we divide the total measurement data into $L>K$ overlapping vectors of length *N*, denoted by $\tilde{\mathbf {b}}_l$, and use them to produce *L* frames $\tilde{\mathbf {u}}_l$.

### Reconstruction algorithm under global motion

**Figure 2:**
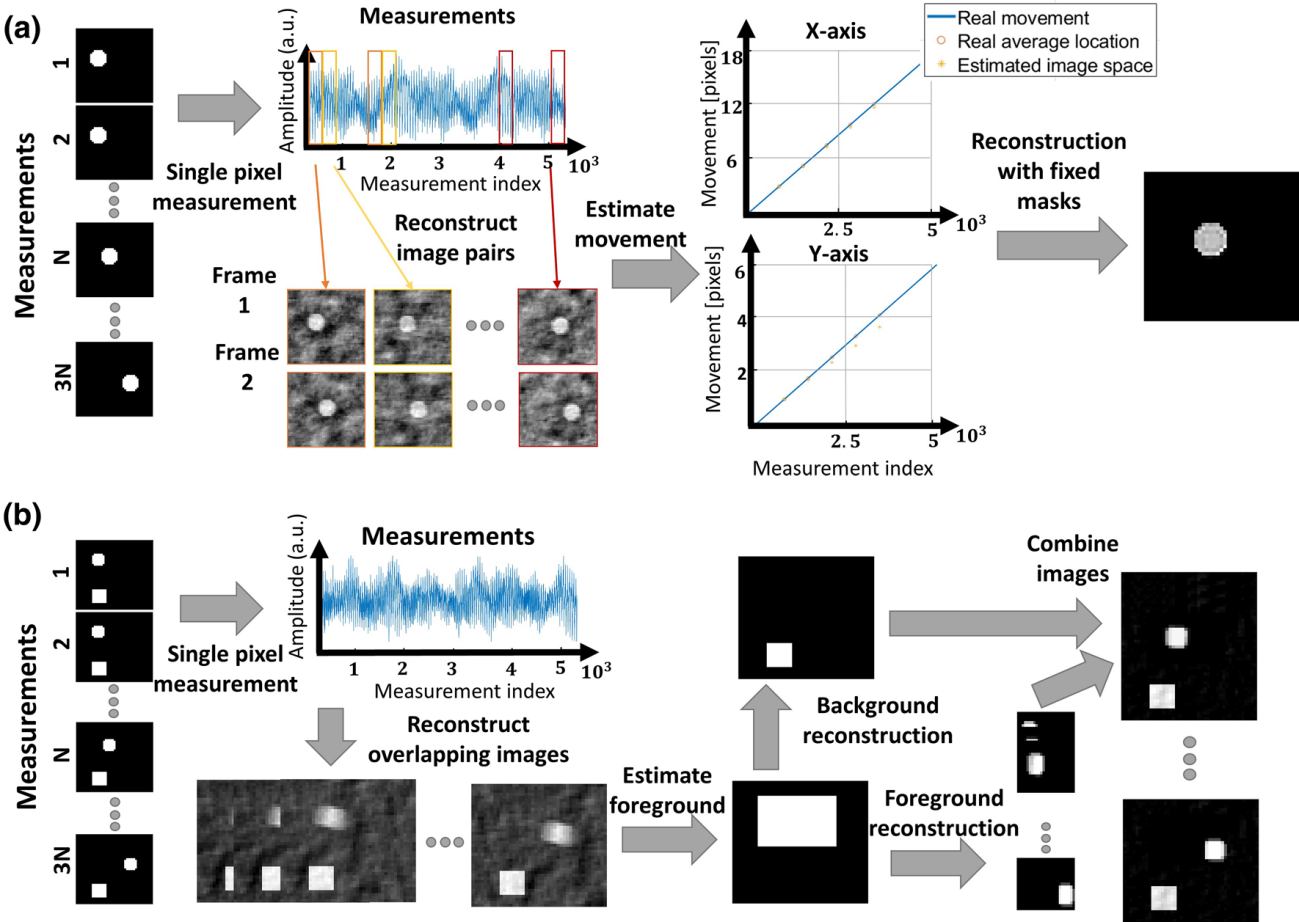
Schematic of the two proposed algorithms (**a**) Flow of the algorithm in case of global motion. (**b**) Flow of algorithm in case of local motion.

In the case of global motion, the entire image moves during the measurement. The algorithm is composed of two main steps, illustrated in Fig. 2a: motion estimation and sampling-matrix adjustment.

Two approaches are proposed for estimating the global motion. In the first approach, the estimation is performed in image space. Accordingly, we arrange the measurement data in *I* subsets of *K* frames, $\mathbf {b}_{k,i}$, and produce the corresponding partial reconstructions, $\mathbf {u}_{k,i}$, using Eq. (3). We then rearrange the elements in the 1D vectors $\mathbf {u}_{k,i}$ to form 2D images, denoted by $\mathbf {U}_{k,i}$. Assuming that $\mathbf {U}_{k+1,i}$ is shifted from $\mathbf {U}_{k,i}$ by *a* and *b* pixels in the *x* and *y* directions, respectively, the values of *a* and *b* may be estimated by maximizing the cross-correlation between the two images:

7 $$\begin{aligned} a^*,b^* = \text {argmax}_{a,b} \sum _{p=0}^{P-1} \sum _{q=0}^{Q-1} \mathbf {U}_{k,i}[p,q] \cdot \mathbf {U}_{k+1,i}[p+a,q+b], \end{aligned}$$

where $\mathbf {U}_{k,i}[p,q]$ denotes the element in the *p* row and *q* column of the matrix $\mathbf {U}_{k,i}$. In practice, since the shift between $\mathbf {U}_{k+1,i}$ and $\mathbf {U}_{k,i}$ may be of sub-pixel, we use an efficient algorithm described in Ref.^30^ to estimate the movement. In the second approach, motion estimation is performed directly in the projection space, rather than in image space. We transform the 1D vectors $\mathbf {b}_{k,i}$ into 2D matrix $\mathbf {B}_{k,i}$ of size $P/I \times Q$. We then use the algorithm of Ref.^30^ to estimate the shift between $\mathbf {B}_{k,i}$ and $\mathbf {B}_{k+1,i}$, whose measurements both originate from the same sampling masks. The shift corresponds to the global movement of the image.

Regardless of whether motion estimation is performed in image or projection space, the procedure is repeated for all pairs of $\mathbf {U}_{k,i}$ and $\mathbf {U}_{k+1,i}$ or $\mathbf {B}_{k,i}$ and $\mathbf {B}_{k+1,i}$, leading to $(K-1)I$ discrete estimates of image motion. These discrete estimates are then interpolated to obtain the continuous motion undergone by the $K-2$ intermediate frames. Since for each frame, the interpolation uses data from both the preceding and subsequent frames, it is not performed for the first and last frames in the data set.

Once the motion between subsequent frames is estimated, it is incorporated into the inversion algorithm. Since the object’s motion is relative to the illumination pattern, the measurement results are the same as those that would be obtained if the object was static and illumination patterns were moving in the opposite direction. Our goal is to reconstruct the vector $\mathbf {u}$, representing the image, from the measurement data $\mathbf {b}$, given the following model:

8 $$\begin{aligned} \mathbf {b} = \mathbf {TS}\mathbf {u}, \end{aligned}$$

where $\mathbf {T}$ is a translation matrix. Each row of the matrix $\mathbf {T}$ corresponds to shifting a different sampling pattern, i.e. different row of $\mathbf {S}$. An explanation and examples for construction of $\mathbf {T}$ can be found at the supplementary Fig. S2.

In the last step we we reconstruct our image. Although our original sampling basis was a full basis, our altered problem is no longer necessarily sampled with a complete basis. We add a TV regularization to the reformulated problem and solve Eq. (4) with an adjusted sampling matrix $\mathbf {TS}$.

### Reconstruction algorithm under local motion

The next algorithm we present, uses separation of different pixels in the image to static background and dynamic foreground. Dividing to background and foreground enables faster frame rate for the foreground reconstruction using less measurements. Figure 2b depicts the algorithm for improving a short video captured with a single-pixel camera.

Assuming our measurements are taken from *K* different frames leading to a total of $K \cdot N$ measurements, we divide the measurements $\mathbf {b}_{\text {tot}}$ to a set of $L > K$, $\mathbf {b}_l$ each containing *N* consecutive measurements, with some overlap between sets of measurements. As discussed in section of subsequent frames, we reconstruct a video $\mathbf {U}_L$ containing *L* frames, using Eq. (2). We use the up-sampled frame rate video to separate static background and dynamic foreground pixels. Background detection may be performed using methods originally developed for videos^31^. In our work we use Gaussian mixture model^32,33^, which detects a foreground mask for each frame. The final single foreground mask is calculated with an OR operator between all foreground masks, and the background mask is calculated by applying a NOT operator on the final foreground mask. Measurements corresponding only to the background pixels are calculated by element-wise multiplication of the image with the background mask and multiplication with the sampling matrix Eq. (1). We then deduce the foreground measurements using the linearity of Eq. (1). In the next step, we reconstruct one background image with a full set of measurements, and multiple foreground images using a small sub-set of measurements, as the foreground images only occupy part of the image. All the reconstructions are obtained by solving an optimization problem with TV regularization Eq. (4). In the final step we blend the foreground images with background image to create a high frame-rate video.

### Evaluation methods

To evaluate our simulation results we compare between our proposed reconstruction and the original image. We use two comparison techniques, the first is root mean square error (RMSE). For two images $\mathbf {U}$ and $\mathbf {V}$, RMSE is mathematically described as:

9 $$\begin{aligned} \text {RMSE} = \frac{1}{N^{0.5}} \left( \sum _{p=1}^P \sum _{q=1}^Q (\mathbf {U}[p,q]-{\mathbf {V}}[p,q])^2 \right) ^{0.5} \end{aligned}$$

The second is structural similarity (SSIM). For two images $\mathbf {U}$ and $\mathbf {V}$, SSIM is mathematically described as^34^:

10 $$\begin{aligned} \text {SSIM} = \frac{(2 \mu _{\mathbf {U}} \mu _{\mathbf {V}} + c_1)(2\sigma _{\mathbf {U} \mathbf {V}} +c_2)}{(\mu _{\mathbf {U}}^2 + \mu _{\mathbf {V}}^2 + c_1) (\sigma _{\mathbf {U}}^2+\sigma _{\mathbf {V}}^2 + c_2)}, \end{aligned}$$

where $\mu _{\mathbf {U}}$ and $\mu _{\mathbf {V}}$ are averages of the two compared images, $\sigma _{\mathbf {U}}^2, \sigma _{\mathbf {V}}^2$ are the variances of the images, $\sigma _{\mathbf {U}\mathbf {V}}$ is the covariance and $c_1,c_2$ are two variables calculated from the dynamic range of image.

## Results

### Numerical simulations

In this section, we demonstrate the performance of our algorithm on simulated data. All simulations were carried out on a Lenovo Ideapad 80TV laptop with 20GB of RAM and an Intel Core i7 2.7 GHz processor, using custom written code in Matlab^35^ software.

#### Image reconstruction under global motion

**Figure 3:**
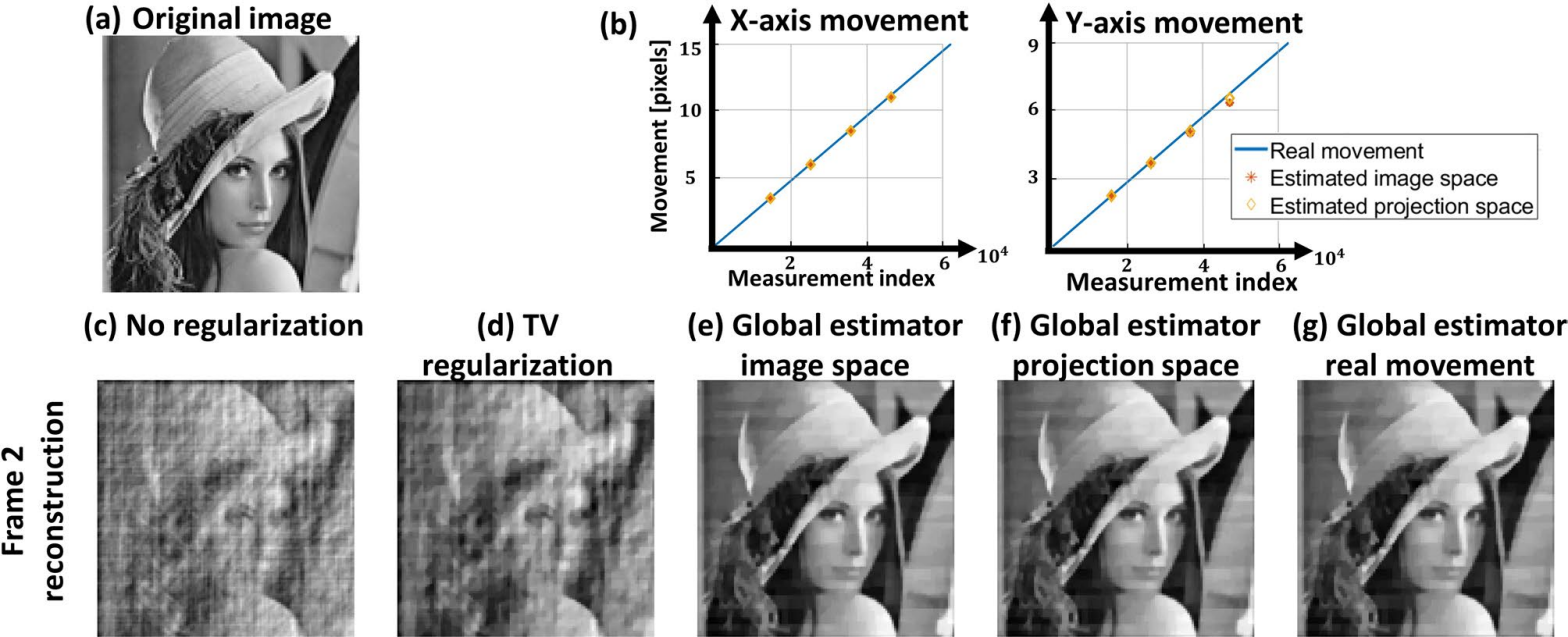
Reconstruction of Lena image undergoing translation at constant velocity. (**a**) The original Lena image. (**b**) Movement of the image during measurements and estimated movement. (**c**–**g**) Reconstruction of the second frame with different reconstruction algorithms.

We demonstrate our algorithm for the case of global motion using the Lena image shown in Fig. 3a and in addition analyze the reconstruction statistics for 10 images obtained from a public image dataset^36^. For all the simulations, we resize the image to $149 \times 151$ pixels, so it fits with corresponding S-matrix containing a total number of $N = 22,499$ pixels. In order to avoid edge effects, we surround the image with a black frame that assures that all the translated versions of the image are covered by the illumination pattern. We simulate three individual frames with a total of 67, 497 measurement points. We performed the reconstruction algorithm using motion estimation in both image space and projection space and compared the results.

**Table 1:** Summary of results of simulations for global-estimator with constant velocity.

	No regularization	TV regularization	Global estimator image space	Global estimator projection space	Global estimator ground-truth
RMSE	0.175	0.155	0.056	0.046	0.04
SSIM	0.17	0.24	0.84	0.85	0.85
Run time [s]	0.3	116	158	133	149

In the first simulation we translate the image on both *x* and *y* axes at constant speed of 5 pixel/frame in x-axis and 3 pixel/frame in y-axis. The movement is continuous, with sub-pixel shifts consecutively applied for each measurement index. A total of three frames were simulated, allowing reconstruction of one frame. The movement of the image as a function of the measurement index is shown in the solid curve in Fig. 3b. Motion estimation was performed with $I=2$, i.e. each frame was divided into two subsets to increase the number of discrete motion estimates. Figure 3 summarizes the reconstruction results obtained under global motion for the second frame. Fig. 3b shows the estimations of the movement in both image and projection space, where the first estimated location is aligned with the real measurement, as we are only interested in relative motion. Projection space estimation achieved a slightly better estimation with average error in position of 0.12 pixels and average error of 0.16 pixels with image space estimation. Figure 3c–g show a comparison of different reconstruction techniques. The images shown are presented without the surrounding black frame. Figure 3c shows the reconstruction obtained with direct inversion formula Eq. (2) using the entire projection set of the second frame, i.e. $N = 22,499$. Figure 3d shows TV regularization reconstruction, where Eq. (4) is solved. Figure 3e–g show reconstruction with our proposed algorithm for global motion, using estimated image space, estimated projection space and real movement data respectively. Evaluation and run time are summarized in Table 1. An additional simulation presenting edge effects on reconstruction appears in the supplementary Fig. S3.

**Figure 4:**
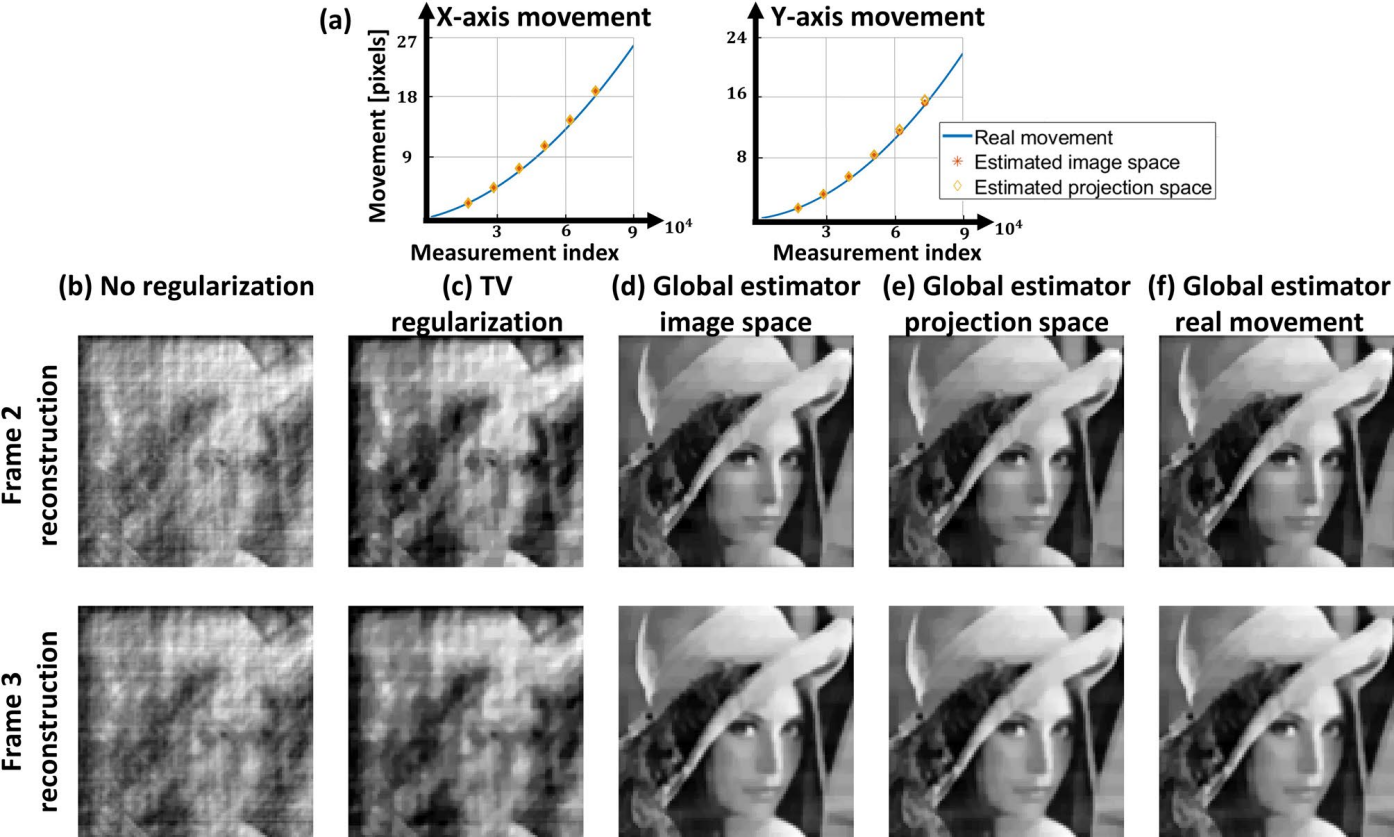
Reconstruction of Lena image undergoing translation with accelerating velocity. (**a**) Movement of the image during measurements and estimated movement. (**b**–**f**) Reconstruction of the second and third frames with different reconstruction algorithms.

**Table 2:** Summary of results of simulations for global-estimator with accelerating image.

	No regularization	TV regularization	Global estimator image space	Global estimator projection space	Global estimator ground-truth
RMSE frame 2	0.176	0.187	0.053	0.087	0.065
RMSE frame 3	0.19	0.2	0.076	0.069	0.073
SSIM frame 2	0.17	0.24	0.85	0.82	0.84
SSIM frame 3	0.16	0.18	0.81	0.82	0.82
Run time [s] frame 2	0.3	143	151	135	129
Run time [s] frame 3	0.3	140	152	134	132

In the second simulation, reconstruction under image acceleration is demonstrated. Translation of the image starts with a speed of 2 pixel/frame in x-axis and 1 pixel/frame in y-axis and the image accelerates in both axes at $2.25 \; \text {pixel/frame}^2$. A total of four frames were simulated and the image reached velocity of 11 pixel/frame in x-axis and 10 pixel/frame in y-axis. From the four simulated frames, we were able to reconstruct two intermediate frames (i.e. frames two and three). Similar to the first simulation, motion estimation was performed with $I=2$. The movement of the image as a function of the measurement index is shown in the solid curve in Fig. 4a. Average error of projection space estimation is 0.6 pixels and for image space estimation is 0.5 pixels. Figure 4b–f shows a comparison of different reconstruction techniques for both frames two and three. Although estimation is not as good as for constant velocity, the reconstructed images with global estimator result in significant improvement. Evaluation and run time are summarized in Table 2.

From these simulation results shown in Figs. 3c–g and 4b–f, it is evident that the movement of the object leads not only to blurring, but also to severe image artifacts. Reconstruction obtained with our global-motion estimator, shows a clear improvement in the image, with only minor differences between the reconstructions obtained with the ground-truth motion and those obtained with our global-motion estimators.

To test the general effect of *I* on the performance of motion-estimation algorithms, we performed image reconstruction on 10 images from a public image database^36^ using different motion parameters and values of *I*. All the simulations were performed with motion estimation in the projection space domain. We report the average pixel estimation error for different velocities and different number of subsets in Table 3. We conducted two simulations with constant velocity and two simulations with acceleration. From our results it is possible to see the trade-off between the number of subsets *I* and estimation performance. For constant velocity, a small number of subsets is preferred, as good estimation requires a large number of measurements, and although reconstructed images suffer from blur, since the velocity is constant the blur effects the compared reconstructed images similarly. However, for scenarios where the velocity changes and especially for rapid changes such as fast acceleration, a small number of subsets results in two images which are blurred by significantly different velocities, resulting in bad estimation. Thus, a larger number of subsets are required to estimate fast changes in velocity.

**Table 3:** Average error in pixel estimation for different velocities and different number of subsets *I*.

	Subset $I=1$	Subset $I=2$	Subset $I=3$	Subset $I=4$	Subset $I=5$
$V_x = 7$ pixel/frame	0.09	0.12	0.23	0.23	0.23
$V_y = 2$ pixel/frame					
$V_x = 10$ pixel/frame	0.32	0.4	0.41	0.55	0.75
$V_y = 5$ pixel/frame					
$a_x = 6 \; \text {pixel/frame}^2$	3.78	7.3	0.39	0.25	0.23
$$a_y = 6 \; \text {pixel/frame}^2$$					
$a_x = 8 \; \text {pixel/frame}^2$	1.8	11.5	0.6	0.66	0.45
$$a_y = 8 \; \text {pixel/frame}^2$$					

#### Image reconstruction under local motion

**Figure 5:**
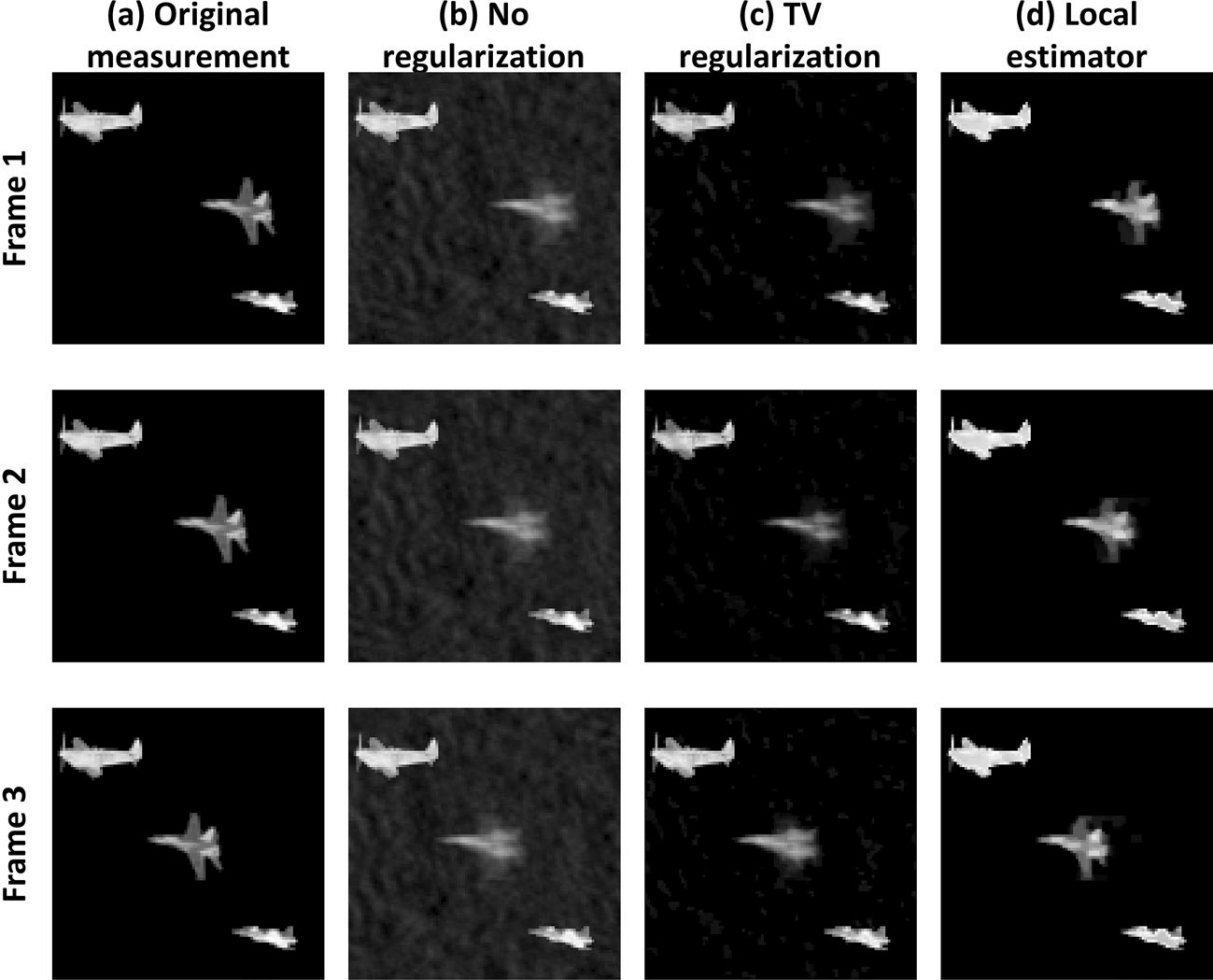
Three frames of a video with local motion. From left to right: the original measurements, reconstruction with matrix inversion Eq. (2), reconstruction with TV regularization Eq. (4), and with our local estimator.

Next, we simulate a video of three airplanes, where two airplanes are part of the static background and one moves 30 pixels during the measurement, as shown in Fig. 5a. We incorporate images from CIFAR10^37^ data-set containing images of size $32 \times 32$. We convert the images to gray scale and interpolate them into an image size of $101 \times 103$, containing $N = 10403$ pixels, with the total simulation resulting in $3 \times N = 31,209$ projection data points. We use our local-motion algorithm for local movement estimation on the entire video. Figure 5b,c respectively show the reconstructions of the three frames using direct Eq. (2), and TV-based Eq. (4) inversion without accounting for the motion of one of the airplanes. While TV regularization significantly reduced the background artifacts in the reconstruction, it still resulted in blurring of the moving airplane. In contrast, the reconstruction performed using our local-motion estimator, shown in Fig. 5d, achieved a clear improvement in image contrast in addition to the reduction in background artifacts. Summary of average results of the three frames is shown in Table 4.

**Table 4:** Summary of results local estimator simulation.

	No regularization	TV regularization	Local estimator
Average RMSE	0.045	0.0347	0.321
Average SSIM	0.32	0.83	0.97
Run time three frames [s]	0.4	493	289

### Experimental setup

The experimental setup consists of three main parts: an illumination system, a detection system and an object. In the illumination system, a red light-emitting diode (LED) illuminates the S-matrix patterns produced on a photo-mask. The patterns are changed by rotation of the photo-mask at a constant speed of 5 Hz. The light transmitted through the photo-mask, which is spatially modulated by the S-matrix patterns, is collected by an objective and is projected on the imaged object. On the other side of the object, a focusing lens is placed to collect the light that goes through the target to a bucket detector (Thorlabs DET 36A - Si photo-detector with a custom trans-impedance amplifier). The photo-detector signal is sampled with an oscilloscope (Keysight DSOX4154A) synchronized with the sequence of projected patterns via the trigger from the rotation stage.

We conducted two kinds of experiments. In the first, we imaged a 1951 USAF resolution target and manually translated it during data acquisition to simulate global motion. In the second experiment, we used a piece of A4 paper with holes punctured by a 125 $\mu$m needle head. The paper was cut into two pieces, where one piece was manually translated and the second piece was stationary to simulate local motion. In both experiments, the manual translation was performed over the y-axis using a mechanical stage (XYF1 - translation mount, Thorlabs). Since movement was performed manually, the velocity was not constant during data acquisition. Data acquisition in both experiments was performed using $347 \times 349$ patterns, containing $N = 121,103$ pixels, with four consecutive frames, with an imaging time of 200 ms per frame.

### Experimental results

Experimental reconstruction were carried out on a desktop with 64GB of RAM and an Intel Core i7 3.5 GHz processor, using custom written code in Matlab software.

#### Global motion

**Figure 6:**
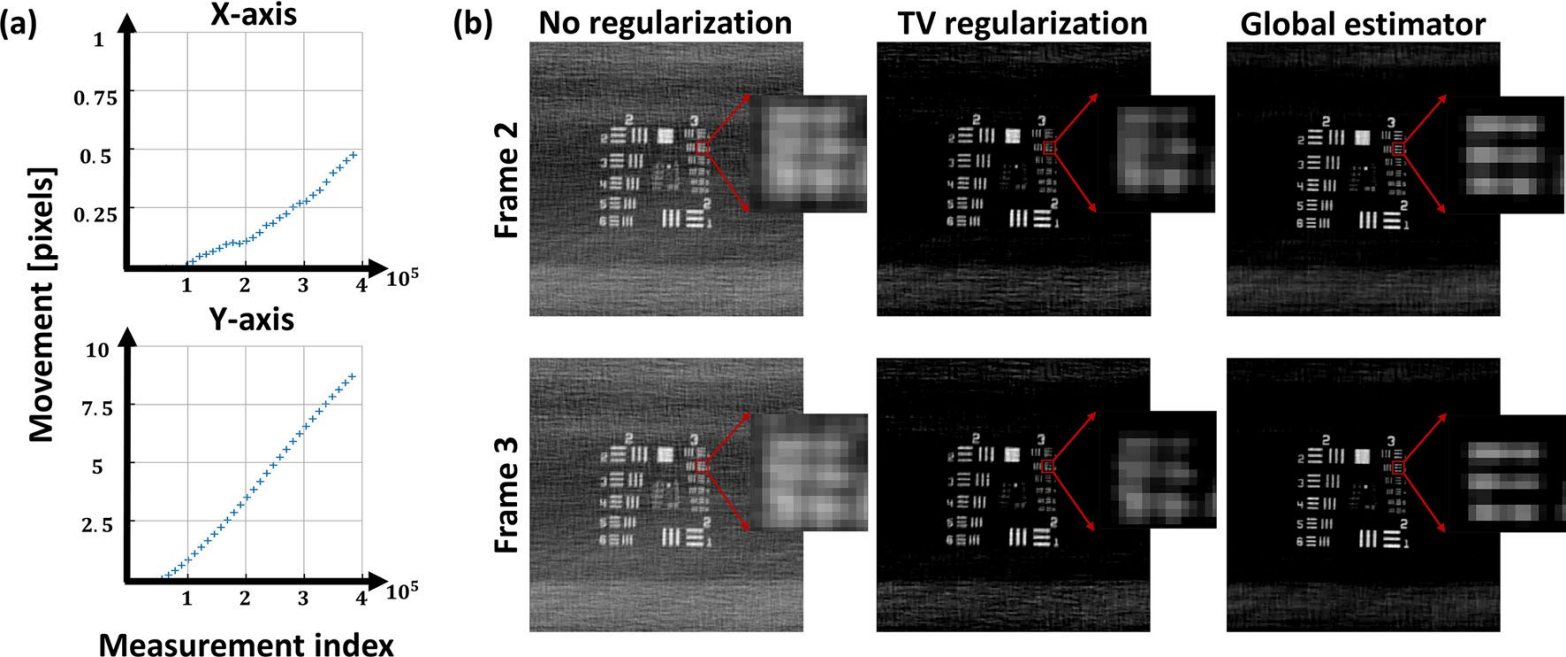
Reconstruction of a moving resolution target. (**a**) Estimated motion of the resolution target. (**b**) From left to right: reconstruction with inversion of S-matrix, TV regularization and global estimator. Zoom in to pattern 3.2 on resolution target, showing improvement.

To reconstruct an image of the USAF 1951 resolution target under global motion, motion estimation was performed in projection space with $I=7$ subsets per frame. Figure 6a shows the estimated motion of the resolution target. The figure shows that while most of the motion was indeed in the y-axis, some residual was also detected in the x-axis Fig. 6b shows a comparison between the reconstruction images using, direct inversion with full data Eq. (2), TV regularization optimization Eq. (4), and our motion-estimation algorithm. The result shows that while the TV constrained reconstruction was successful in reducing the image artifacts due to the motion, it could not overcome the blurring. In contrast, the estimated motion was used in the reconstruction, significant contrast enhancement was obtained. Comparison of RMSE and SSIM for the average result of the two frames is shown in Table 5.

**Table 5:** Summary of results global estimator experiment.

	No regularization	TV regularization	Global estimator
Average RMSE	0.134	0.078	0.059
Average SSIM	0.22	0.8	0.84

#### Local motion

**Figure 7:**
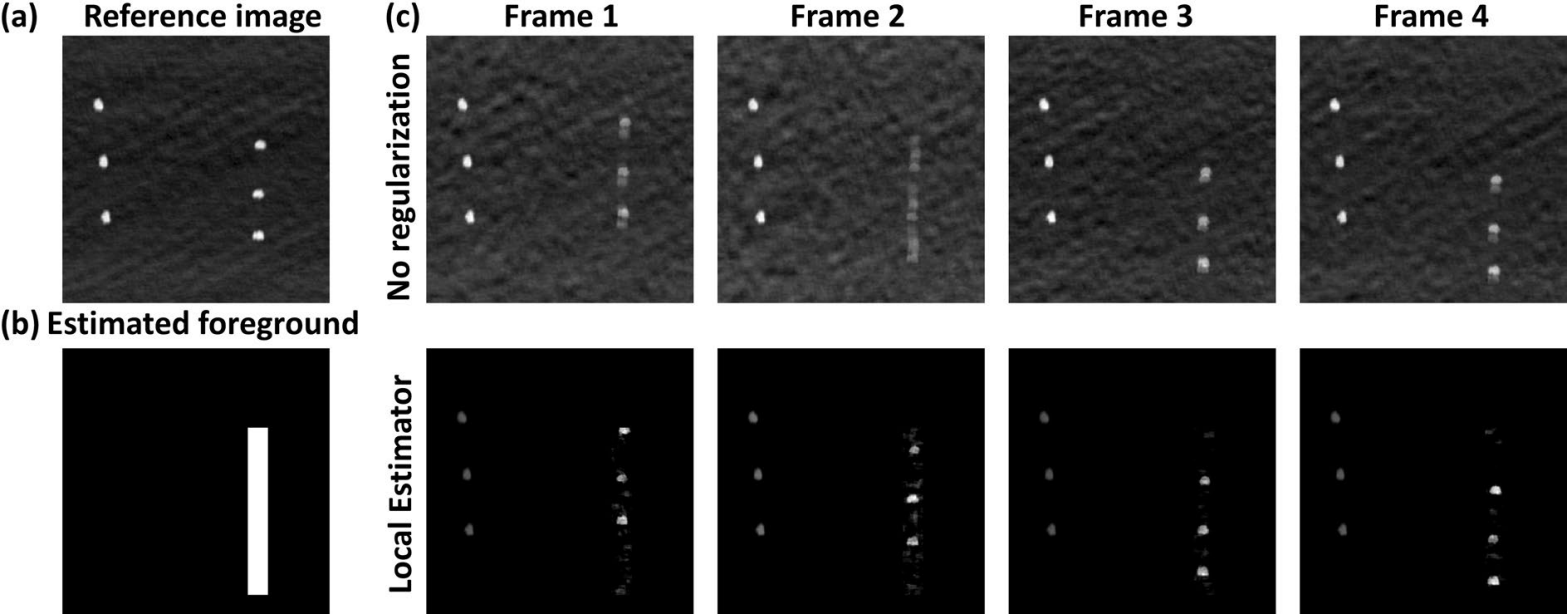
Reconstruction of a moving target with static background. (**a**) Is a reference image taken with stationary points. (**b**) Estimated foreground of moving parts. (**c**) First row is reconstruction using inverse s-matrix. Second row is reconstruction using local estimator algorithm.

The results of the local-motion experiment are shown in Fig. 7. Figure 7a shows the reference image, obtained before the right side of the target was manually translated. Figure 7b shows the foreground of the moving part of the target, estimated by our algorithm. Figure 7c shows a comparison between the direct reconstructions, obtained using Eq. (2) with full data (top panel), and the reconstructions obtained by our local motion-estimation algorithm (bottom panel). The comparison is shown for all four frames. To calculate the RMSE and SSIM of the reconstructions, the right sides of the reconstructed images were shifted up to be realigned with the right side of the reference image. A comparison of the RMSE and SSIM for the average result of the four frames is shown in Table 6.

**Table 6:** Summary of results local estimator experiment.

	No regularization	Local estimator
Average RMSE	0.22	0.10
Average SSIM	0.12	0.48

## Conclusion

In this paper we developed a new algorithmic approach to reduce the effect of motion in SPI. Our approach is based on estimating the motion between consecutive frames and integrating it into the model matrix of the SPI measurement. By using a cyclic model matrix, global motion of the imaged object may be estimated from the measured data (projection space), without performing any image reconstruction, leading to a numerically efficient algorithm. We tested our algorithms on both numerical and experimental data and demonstrated significant improvement in reconstruction quality in comparison to conventional algorithms that assume a static image.

In the Supplementary Information, additional simulations are provided in which the projections were calculated with a non-cyclic model matrix, namely with a randomized Hadamard basis, where the reconstructions were produced using both full-data inversion Eq. (2) and CS Eq. (4). The use of a randomized basis enabled us to use more efficiently CS reconstruction algorithms for each frame, thus reducing the number of data points required per frame. However, the use of a non-cyclic basis required performing the motion estimation in image space, resulting in larger reconstruction complexity.

## Discussion

While our algorithms were developed for piece-wise linear motion in 2D, several extension are readily possible. For example, comparison of subsequent frames can also detect 3D motion as long as the through-plane motion does not lead to image defocusing. In that case, algorithms that quantify scaling and shear, e.g. correlation methods based on Fourier-Mellin transformation^38–40^, can be used to estimate the 3D motion of the target. This proposed extension can also be used to quantify in-plane rotation, which was not treated in our work.

Our results show that motion correction is important for any application in which the object is even slightly dynamic since even a few-pixel motion during the acquisition of a single frame can lead to significant reconstruction artifacts. Our global-motion algorithm may be used in cases in which there is relative motion between the camera and target and may benefit applications such as remote sensing^3^ and single pixel telescope^4^. Additional applications for global-motion correction may be found in the medical field. For example, in the case of SPI ophthalmoscopes, eye movement during imaging was considered one of the limitations for *in vivo* applications^10^. Our local-motion algorithm may be used for imaging dynamic objects moving over a relatively static background. Specific application include fluorescence microscopy^11^ and two photon microscopy^12–14^. In such applications, merely distinguishing between the background and foreground can significantly reduce the size of the the region that needs to be updated between subsequent frames, enabling significant acceleration in image acquisition.

## Supplementary Information

Supplementary Information.

## Data Availability

The data that support the findings of this study are available from the corresponding author upon reasonable request.
