# Supplementary material for: Single-pixel imaging of dynamic objects using multi-frame motion estimation
Source: Sci Rep. 2021 Apr 8;11:7712. doi: 10.1038/s41598-021-83810-z (PMC8032706; doi:10.1038/s41598-021-83810-z)
Supplement: Supplementary file 1 — Supplementary Information. [file 41598_2021_83810_MOESM1_ESM.pdf]

# Single-pixel imaging of dynamic objects using multi-frame motion estimation

**Sagi Monin[1], Evgeny Hahamovich[1], and Amir Rosenthal[1,*]**

[1]Department of Electrical Engineering, Technion – Israel Institute of Technology, Haifa, 32000, Israel
[*]amir.r@ee.technion.ac.il

**Supplementary - Utilizing cyclic sampling matrix**

Generally, motion estimation must be performed in image space. However, in the special case of a cyclic sampling matrix, we explain how the cyclic nature of the sampling matrix may be exploited to estimate motion without image reconstruction.

**Cyclic matrix as circular convolution**

Cyclic matrices, where each row is a circular shift of the previous row, describe input-output behavior of one-dimensional linear periodic systems. From this theory, we can rewrite our sampling process as:

$$\mathbf{b} = \mathbf{s}_1 \circledast \mathbf{u}, \tag{1}$$

where $\mathbf{u}$ is a row stack of the image, $\mathbf{s}_1$ is the first row of the sampling matrix $\mathbf{S}$, and $\circledast$ is a cyclic convulsion operator.

**1D correlation**

From Eq.1 and associativity of convolution we get that the shift of a row stack image $\mathbf{u}$ results in the same shift for the measurement vector $\mathbf{b}$:

$$\mathbf{s}_1 \circledast \mathbf{u}[q-c_0] = \mathbf{s}_1 \circledast (\mathbf{u}[q] * \delta[q-c_0]) = (\mathbf{s}_1 \circledast \mathbf{u}) * \delta[q-c_0] = \mathbf{b} * \delta[q-c_0] = \mathbf{b}[q-c_0], \tag{2}$$

where $c_0$ is the shift of the original vector $\mathbf{u}$. Thus, if we estimate the shift between two measurement vectors, we can deduce the shift between the two row-stack vector images.

**Shift of 2D images as 1D signals**

Since the motion of the image is not a cyclic shift, we limit ourselves to zero-padded images and ignore edge effects. For any 2D image (matrix), we can write a row-stack vector of the image, where we concatenate the rows of the image to a vector.
Claim: For a zero-padded image $\mathbf{U} \in \mathbb{R}^{P\times Q}$ a shift of $c_0$ and $d_0$ in the $x$ and $y$ axes respectively, will result in a shift of $c_0 + d_0 \cdot Q$ for the row-stack vector.
Proof: We write the row-stack vector of the image $\mathbf{U}_1$, as: $\mathbf{u}_1[q+Q\cdot p] = \mathbf{U}_1[p,q]$. The shifted image is described as: $\mathbf{U}_2[p,q] = \mathbf{U}_1[p-d_0, q-c_0]$. Hence, we can write:

$$\mathbf{u}_1[q+Q\cdot p-(c_0+Q\cdot d_0)] = \mathbf{u}_1[q-c_0+Q\cdot(p-d_0)] = \mathbf{U}_1[p-d_0, q-c_0] = \mathbf{U}_2[p,q] = \mathbf{u}_2[q+Q\cdot p] \tag{3}$$

From Eq. 3 we see, that a shift of $c_0$ and $d_0$ in the 2D image results in a shift of the 1D row-stack vector of $c_0 + d_0 \cdot Q$.

**2D projection images for motion estimation**

In the previous sub-section we showed that shift of 2D images results in shift of their 1D row-vector representation. This claim is applicable in the reverse direction as well, shift in 1D signals results in a shift of the 2D images. We use this property to rearrange our measurements to 2D matrices and estimate motion directly on projection space.

$$\mathbf{B} = \tilde{\mathbf{S}} \circledast \mathbf{U}, \tag{4}$$

where $\tilde{\mathbf{S}}$ is the convolution matrix (rearranged as an image from $\mathbf{s}_1$), and $\mathbf{B} \in \mathbb{R}^{P\times Q}$ is our sampled measurements in a matrix form, we denote this matrix as projection space. The matrix form of the samples $\mathbf{B}$, can also be formulated using Eq. (1) in the manuscript to obtain a vector form, and rearranging to a matrix with $\mathbf{B}[p,q] = \mathbf{b}[q+p\cdot Q]$. We use the projection matrix to estimate motion between measurements taken from different frames. We also use only part of the projection space to estimate spatial changes between two shifted images.

It is important to note that we do not claim that cross-correlation maps for 2D images and 1D signals are equal. We only claim that the shift in 2D images can be mapped to 1D signals, and maximum of cross-correlation will result in the same location.

**Supplementary figure S1 - S-matrix arrangement**

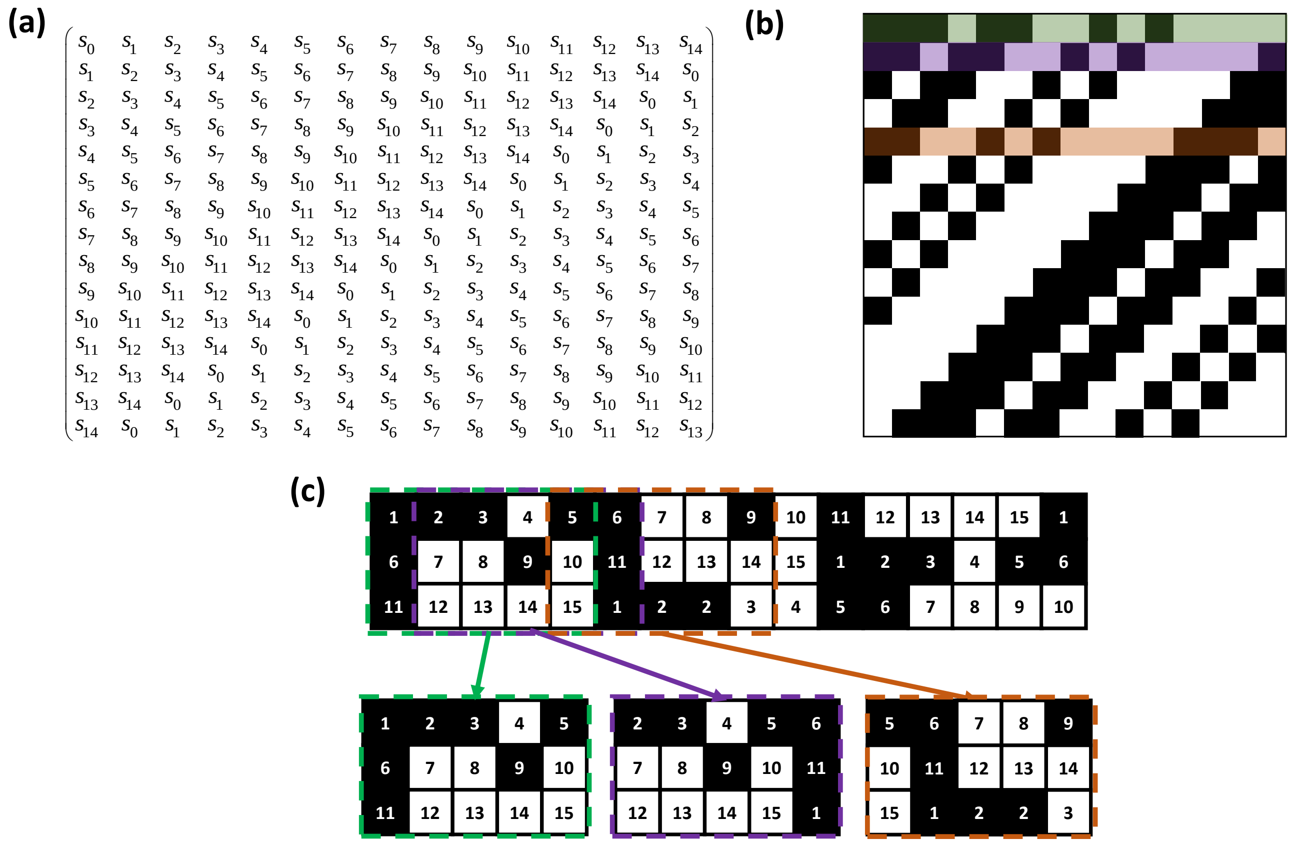


**Figure 1.** **S**-matrix and its arrangement as sampling mask. (a) **S**-matrix of order N=15. (b) Values of **S**-matrix, white color represents 1’ and black color represents 0’. The color of the highlighted rows in the matrix correspond to the different patterns encircled with different colors. (c) The pixel arrangement of **S**-matrix on the sampling mask, and three different sampling patterns.

**Supplementary figure S2 - Translation matrix example**

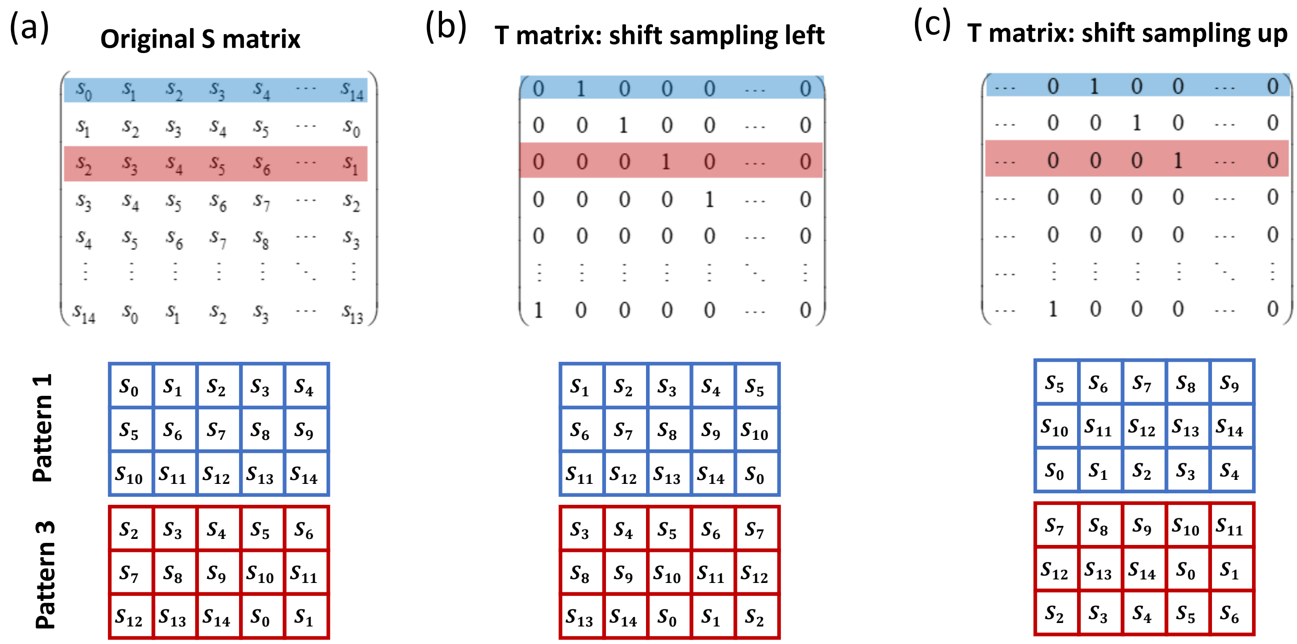


**Figure 2.** (a) Original **S**-matrix with N=15 and two-dimensional masks. (b) **T** matrix shifting all masks one pixel to the left. Each row of the translation matrix shifts a different mask. (c) **T** matrix shifting all masks one pixel up. The matrix column starts from the $5^{th}$ index.

The translation matrix is initialized with the identity matrix: $\mathbf{T} = \mathbf{I}$. After estimating the movement with global-estimator algorithm, we then loop over each row of the matrix and shift it to the right (or left) a number of entries equal to the estimated shift. If the $i^{th}$ measurement corresponding to the $i^{th}$ image was shifted $c$ pixels in the $x$ axis in comparison to the first image, then we cyclic shift the entry in the $i^{th}$ row of the translation matrix by $c$ pixels to the right. In case of a shift of $d$ pixels in $y$ axis of the image, we shift the entry by $d \times Q$ where $Q$ is the number of columns of the image, and in case of sub-pixel shifts we use linear interpolation to shift the entry.

In Fig. 2, an example for either shifting all sampling patterns one pixel to the left or one pixel up is given. Each row of the translation matrix alters a different sampling pattern.

**Supplementary figure S3 - Global motion simulation without zero-padding**

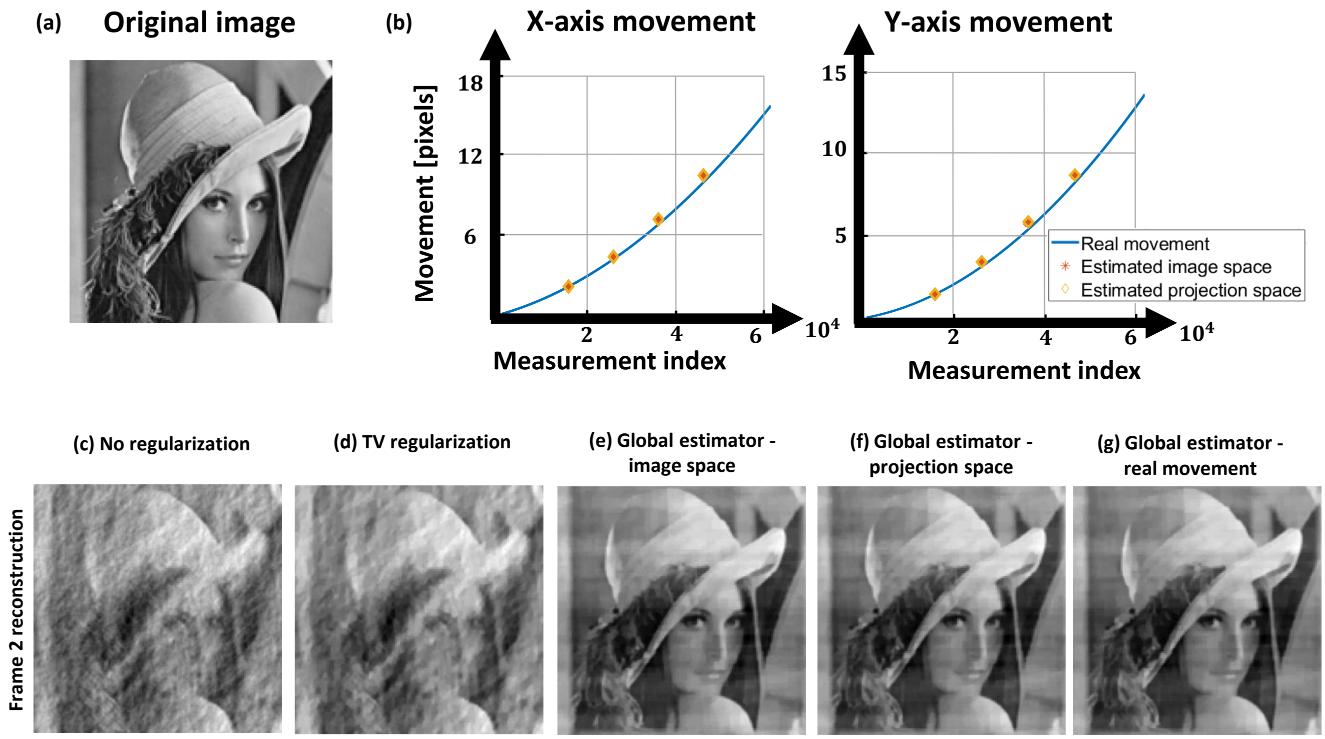


**Figure 3.** Reconstruction without zero-padding. (a) The original Lena image. (b) Movement of the image during measurements and estimated movement. (c-g) Reconstruction of the middle frame with different reconstruction algorithms.

For no padding simulation presented in Fig. 3 we use the same parameters presented in Fig.(4) of the main manuscript, with three frames are simulated allowing reconstruction of a single frame. The starting velocities are 2 pixel/frame in x-axis and 1 pixel/frame y-axis and the image is accelerating in both axis at 2.25 pixel/frame$^2$. Without zero-padding the image, this simulations emphasises edge effects on the image. The image is resized to $(149+18)\times(151+18)$ pixels and for each measurement the center of size $149\times151$ is cropped and sampled. In this simulation we can see edge effects resulting from lost information which do not appear in the previous simulation. Summary of simulation results is depicted in Fig. 3. From the simulation we can see the deterioration of the results in all reconstructions stemming from information lost at the edges. The deterioration does not appear to damage the fine details in the image, but create smearing effects. Motion estimation was done by dividing the frames to subset of two images ($I = 2$). Evaluation and run time are summarized in table 1.

| | No regularization | TV regularization | Global estimator image space | Global estimator projection space | Global estimator ground-truth |
|---|---|---|---|---|---|
| RMSE | 0.163 | 0.136 | 0.1 | 0.094 | 0.094 |
| SSIM | 0.21 | 0.35 | 0.71 | 0.74 | 0.74 |
| Run time [s] | 0.3 | 78 | 96 | 126 | 90 |

**Table 1.** Summary of results of simulations without zero-padding.

**Supplementary figure S4 - Global motion simulation with Hadamard patterns**

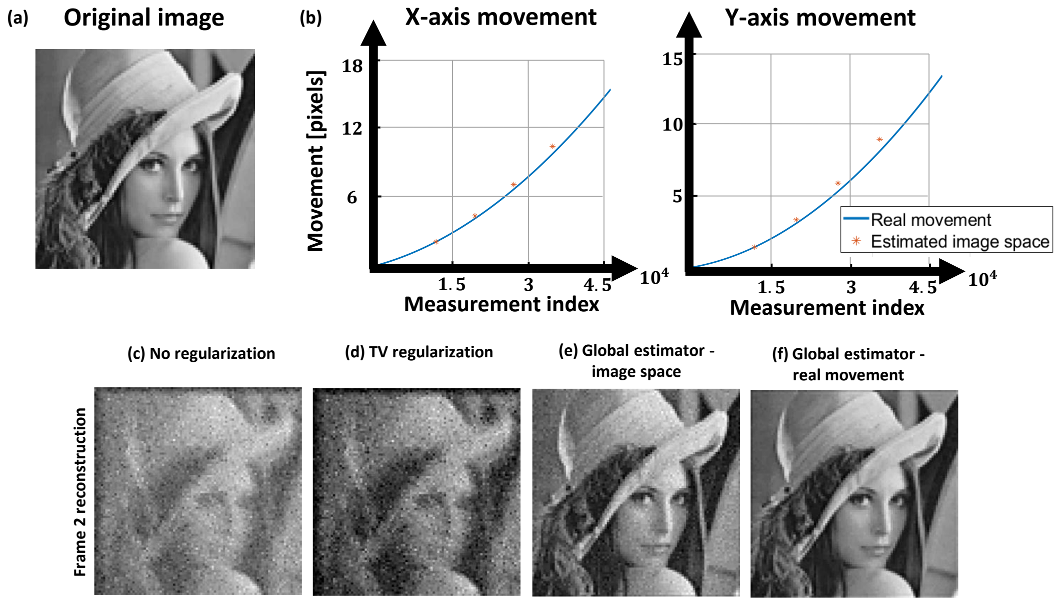


**Figure 4.** Reconstruction of Lena image undergoing translation at accelerating velocity, sampled with Hadamard patterns. (a) The original Lena image. (b) Movement of the image during measurements and estimated movement. (c-f) Reconstruction of the second frame with different algorithms.

We use a randomized-ordered Hadamard pattern to with n=16384 elements, to simulate use of Hadamard pattern. In the presented Hadamard simulation the velocity and acceleration are similar to S-matrix simulation presented in Fig.(4) of the main manuscript, with three frames are simulated allowing reconstruction of a single frame.. The starting velocities are 2 pixel/frame in x-axis and 1 pixel/frame y-axis and the image is accelerating in both axis at 2.25 pixel/frame$^2$. Motion estimation was done by dividing the frames to subset of two images ($I = 2$). Evaluation and run time are summarized in table 2.

| | No regularization | TV regularization | Global estimator image space | Global estimator ground-truth |
|---|---|---|---|---|
| RMSE | 0.17 | 0.17 | 0.03 | 0.02 |
| SSIM | 0.22 | 0.22 | 0.88 | 0.94 |
| Run time [s] | 0.22 | 70 | 251 | 228 |

**Table 2.** Summary of results with Hadamard acquisition simulation.

**Supplementary figure S5 - Global motion simulation with Compressed sensing**

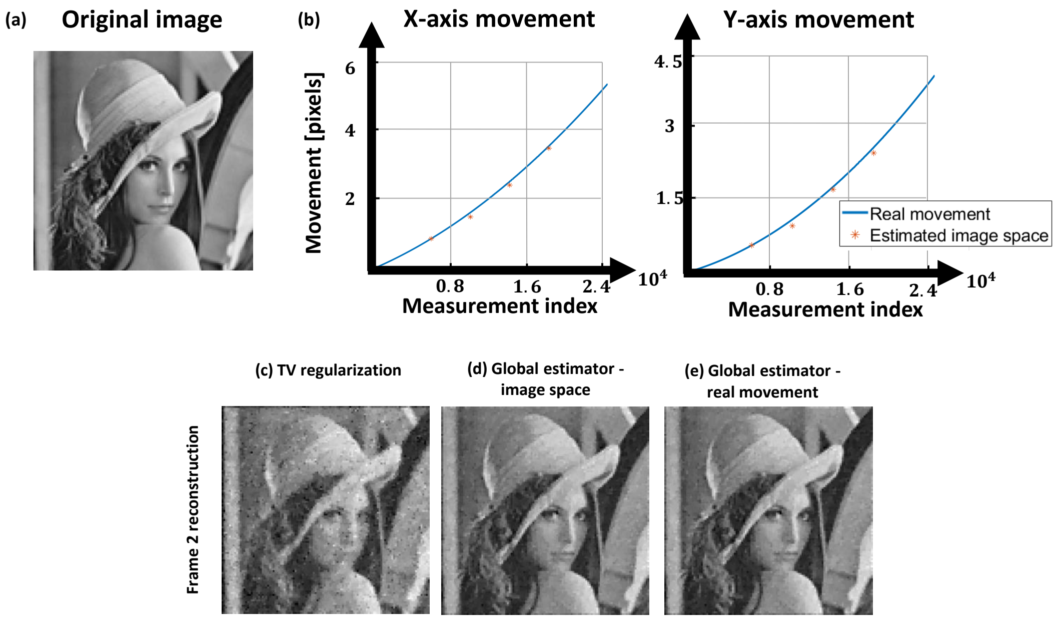


**Figure 5.** Reconstruction of Lena image undergoing translation with accelerating velocity, with 50% of samples. (a) The original Lena image. (b) Movement of the image during measurements and estimated movement. (c-e) Reconstruction of the second frame with different algorithms.

A simulation with randomized-ordered Hadamard pattern, simulating acquisition of only 50% of samples per frame is presented in Fig. 5. The velocity and acceleration are similar to S-matrix simulation presented in Fig.(4) of the main manuscript, with three frames are simulated allowing reconstruction of a single frame. The starting velocities are 2 pixel/frame in x-axis and 1 pixel/frame y-axis and the image is accelerating in both axis at 2.25 pixel/frame$^2$. Since the velocities are similar to previous simulations, and only 50% of patterns are acquired the total movement of the image is smaller. Motion estimation was done by dividing the sub-sampled frames to subset of two images ($I = 2$). For image reconstruction in motion estimation step we use a TV solver (TVAL). Evaluation and run time are summarized in table 3.

| | TV regularization | Global estimator image space | Global estimator ground-truth |
|---|---|---|---|
| RMSE | 0.1 | 0.047 | 0.044 |
| SSIM | 0.46 | 0.81 | 0.82 |
| Run time [s] | 367 | 128 | 93 |

**Table 3.** Summary of results with 50% acquisition simulation.
